# Integrating Seniors Centres Into Primary Healthcare: A Community-Engaged Approach to Collaborative Care

**DOI:** 10.1093/geroni/igaf122.339

**Published:** 2025-12-31

**Authors:** Mei Lan Fang, Rebecca White, Anthony Kupferschmidt, Michael Volker

**Affiliations:** Simon Fraser University, Vancouver, British Columbia, Canada; Simon Fraser University, Vancouver, British Columbia, Canada; City of Vancouver, Vancouver, British Columbia, Canada; 411 Seniors Centre Society, Vancouver, British Columbia, Canada

## Abstract

This presentation highlights the vital role of seniors centres as third places in promoting older adults’ well-being and social connectivity, yet their integration into primary healthcare is often overlooked. This project aims to foster collaboration between seniors centres and healthcare organizations to co-develop a community-integrated health model that enhances healthcare access, social inclusion, and well-being for older adults. Through five cross-sectoral workshops rotated across senior centre locations, we engaged representatives from four Vancouver-based seniors centres through the Alliance of Seniors Centres and four healthcare organizations—Providence Health Care, Vancouver Division of Family Practice, Vancouver Coastal Health, and Mid-Main Community Health Centre. Guided by community-engaged research principles, the workshops focused on a future research and practice agenda, long-term goals, and identifying conditions for success with measurable impact indicators toward a Theory of Change framework. A key engagement feature was the intergenerational pairing of seniors with students, fostering cross-generational learning, research inclusion, and community ties. Findings were analyzed using a thematic framework approach, and key themes were validated across stakeholder groups to ensure relevance in shaping integrated healthcare models. Preliminary themes include improved collaboration between seniors centres and healthcare providers, the role of third places in supporting healthy aging outcomes, and establishing a sustainable research agenda. A key implication involves opportunities to advance community-based research by reshaping healthcare delivery to be more inclusive, accessible, and responsive to older adults. Findings will inform policymakers, practitioners, researchers, and seniors centre staff and Board members, offering actionable strategies for integrating seniors centres into primary healthcare.

